# Phenotyping for Genetic Improvement of Feed Efficiency in Fish: Lessons From Pig Breeding

**DOI:** 10.3389/fgene.2018.00184

**Published:** 2018-05-24

**Authors:** Pieter W. Knap, Antti Kause

**Affiliations:** ^1^Genus-PIC, Schleswig, Germany; ^2^Biometrical Genetics, Natural Resources Institute Finland (Luke), Jokioinen, Finland

**Keywords:** fish, pigs, genetic improvement, feed efficiency, feed intake, body composition, maintenance costs, residual feed intake

## Abstract

Feed incurs most of the cost of aquaculture production, so feed efficiency (FE) improvement is of great importance. Our aim is to use work done in pigs to formulate a logical framework for assessing the most useful component traits influencing feed intake (FI) and efficiency in farmed fish – either to identify traits that can together be used for genetic improvement of FE, or as substitute traits for FI recording. Improvement of gross FE in growing fish can be accomplished by selection for increased growth rate. However, the correlation of growth with FE is typically only modest, and hence there is room for further improvement of FE through methods other than growth selection. Based on a literature review we propose that the most effective additional methods are selection for reduced body lipid content and for reduced residual FI (RFI). Both methods require more or less sophisticated recording equipment; in particular, the estimation of RFI requires recording of FI which is a challenge. In mammals and birds, both these approaches have been effective, and despite the high costs of FI recording, the RFI approach can be cost-efficient because maintenance requirements are high and therefore RFI variation covers a large part of FI variance. Maintenance requirements of fish are lower and therefore RFI variation covers a smaller part of FI variance. Moreover, accurate high-volume routine individual FI recording is much more challenging in fish than in mammals or birds. It follows that selection for reduced body fat content is likely a more effective (and certainly more cost-efficient) way to improve feed conversion ratio in fish than selection for reduced RFI. As long as body fat content is dealt with as an explicit selection criterion, the only valid reason for FI recording would be the requirement of RFI reduction. So, if RFI reduction is not required, there would be no need for the expense and effort of individual FI recording – and in fish breeding that would be a very desirable situation. Solid evidence for these propositions is still scarce, and their generality still needs to be confirmed.

## Introduction

Feed efficiency (FE) is one of the most economically important traits for the aquaculture supply chain (e.g., Besson et al., 2014; Kankainen et al., 2016) as it is in terrestrial livestock production (e.g., Knap and Wang, 2012). Improved efficiency also means that more nutrients are converted into fish tissues, reducing the nutrient load to the environment. However, the current novel fish feeds that include large amounts of plant-based ingredients typically reduce FE, emphasizing that efficiency needs to be further improved on the novel diets (Quinton et al., 2007a; Hardy, 2010).

Although the first study on the genetics of FE in farmed fish is old (Kinghorn, 1983), its progress lags behind that in terrestrial livestock animals, especially pigs and poultry (Knap and Wang, 2012; Willems et al., 2013). One reason is that the recording of feed intake (FI) on individual fish held in schools and under commercial conditions still remains a challenge (Kause et al., 2006a). Moreover, in many cases, the studies on aquaculture genetics are isolated from the progress made with mammals and birds. Although being biologically different, animals farmed for meat production, such as pigs, fish, poultry, and beef cattle, do share common topics and methodology, and the progress made in mammals and birds potentially aids the progress to be made in aquatic species. For instance, in pigs, a successful approach has been to assess the factors causing variation in FE and then using these component traits to improve the composite trait by breeding (Clutter, 2011; Knap and Wang, 2012).

Our present aim is to use the work done in pigs to formulate a logical framework for assessing the most useful component traits influencing FI and efficiency in farmed fish – either in order to find several traits that can together be used for genetic improvement of FE, or as substitute traits for FI recording. The growth of protein, lipid, and structural tissues demands different amounts of energy and FI, and impacts wet weight growth differently (Cho and Kaushik, 1990; Emmans, 1994; Jobling, 1994; Kause et al., 2016). Moreover, high metabolic maintenance costs reduce efficiency in endotherms (Knap, 2008; Knap and Wang, 2012; Gilbert et al., 2017) but not in fish. We therefore argue that in farmed fish, body composition is likely to be a more promising component trait of FE than metabolic maintenance costs are.

## Basic Concept

When livestock and aquaculture breeding is directed to FE, the obvious breeding objective trait is *gross* FE: the amount of output (e.g., meat) per amount of input (i.e., feed), or vice versa. This is what the producers are interested in. The output is commonly measured as body weight (kg), or alternatively, as energy (kJ) or protein mass (kg).

In growing animals including farmed fish, the most commonly used gross FE traits are the feed conversion ratio (FCR), calculated as kilogram feed consumed divided by kilogram body weight gained (or sometimes carcass weight gained), or its reverse, FE. It follows that there are only two ways to improve FCR and FE: (i) by increasing growth rate (GR), and (ii) by reducing FI.

## The Component Traits

In pig breeding, the logic has been to separate FI into its underlying component traits which can all be used in a selection index to improve FE. The same approach is applicable to fish.

The FCR denominator component GR is by far the most common selection trait in breeding programs for growing animals; it is easily recorded *in vivo*. By contrast, the measurement of the numerator component FI is much more difficult and costly, particularly in group-housed animals where the FI of an individual is of interest.

In farmed fish, heritability estimates for FE traits average around 0.14 (**Table 1**), implying potential for genetic improvement. However, the estimates are lower than in terrestrial livestock species with average heritabilities for FE traits of 0.25 in beef cattle (Pitchford, 2004; Berry and Crowley, 2014) and 0.3 in pigs (Knap and Wang, 2012).

**Table 1:** Heritability estimates for feed efficiency in farmed fish.

Species	Trait	Mean *h*^2^ (range)	Source
European whitefish (*Coregonus lavaretus*)	FE	0.065 (0.06–0.07)	Quinton et al., 2007a
Rainbow trout (clones) (*Oncorhynchus mykiss*)	RFI	0.23	Grima et al., 2008
Rainbow trout	FCR	0.12 (0.10–0.13)	Kause et al., 2016
Rainbow trout	RFI	0.13 (0.11–0.14)	Kause et al., 2016

*FE, feed efficiency; FCR, feed conversion ratio; RFI, residual feed intake.*

In most fish species, selection for rapid growth is expected to improve FCR as a correlated genetic response (Quinton et al., 2007a; Kause et al., 2016; De Verdal et al., 2017a), similar to terrestrial animals. Yet, in selection experiments for rapid growth, the correlated change in FE can be very minor compared to the direct genetic change, making the detection of a selection response difficult (Sanchez et al., 2001; Mambrini et al., 2004; De Verdal et al., 2017a).

In growing pigs, GR and FI have a moderately high heritability: Clutter’s (2011) review gives 0.03–0.49 (average 0.29) for GR, and 0.13–0.62 (average 0.29) for FI. By contrast, the heritability estimates obtained for FI in fish (**Table 2**) are lower and range from 0 to 0.41 (average 0.19). The low heritability is mainly due to high residual variance rather than low genetic variance (Kause et al., 2006a,b, 2016). The high residual variance results from a large day-to-day FI variation in fish; this complicates the recording of long-term FI by methods that are based on daily snapshots. The x-ray method can be used to record individual FI in fish reared under commercial conditions in schools (Talbot and Higgins, 1983; Kause et al., 2006a): feed pellets are enriched with glass ballotini beads, and the x-ray of a fish reveals the amount of feed consumed during a single day. Video recording can be used to count the number of feed pellets eaten in a day by individual fish held in individual aquaria (De Verdal et al., 2017b). Both methods are laborious, and many repeated measures of daily FI are needed to achieve high recording accuracy and a long enough recording period (Kause et al., 2006a; De Verdal et al., 2017b).

**Table 2:** Heritability estimates for feed intake in farmed fish.

Species	Mean *h*^2^ (range)	Source
Channel catfish (full-sib design) (*Ictalurus punctatus*)	0.39 (0.37–0.41)	Silverstein et al., 2001
Rainbow trout	0.10 (0.00–0.19)	Kause et al., 2006a
European whitefish	0.27 (0.21–0.32)	Quinton et al., 2007a
Rainbow trout (clones)	0.11	Grima et al., 2008
Rainbow trout	0.10 (0.09–0.11)	Kause et al., 2016

The question is then to what extent the difficult and costly FI measurement can be usefully replaced by measurement of any underlying traits that are easier and cheaper to record. Three such traits are commonly recognized, as follows.

**FIGURE 1:**
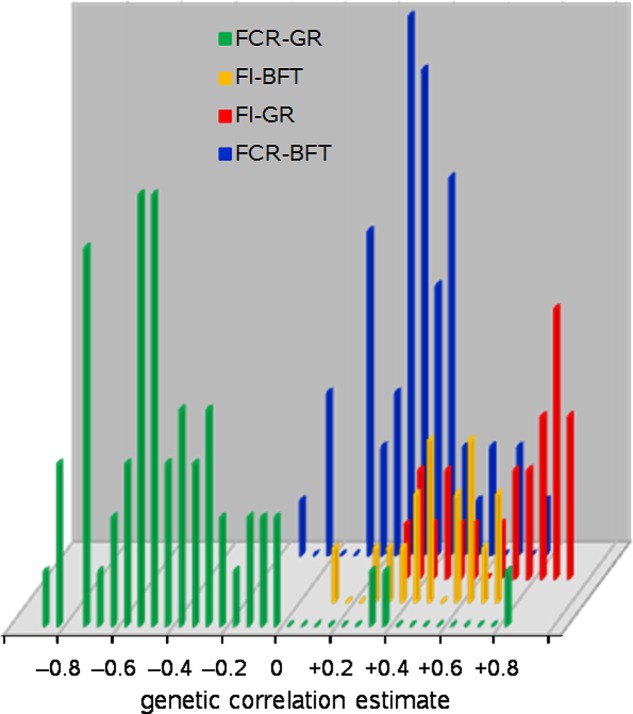
Frequency distributions of genetic correlation estimates of feed intake (FI) and feed conversion ratio (FCR) with growth rate (GR) and backfat depth (BFT) in growing pigs; 154 estimates from 55 literature sources.

First: GR itself is the most obvious correlate: more growth requires more nutrients. Nutrient availability for growth depends not only on gross FI but also on digestion, absorption, and partitioning processes. These vary across animal species, with animal age, feed composition, and animal management and health settings. Hence the correlation between GR and FI is positive but lower than unity; see **Figure 1** where the genetic correlations between these traits in growing pigs range from +0.27 to +0.89.

Second: animal growth is the result of changes in body protein, lipid, water, and mineral content. Deposition of 1 g of lipid leads to 1.1 g wet weight gain including 0.1 g water in the associated adipose tissue, whereas deposition of 1 g of protein leads to 4–5 g wet weight gain including 3–4 g water (Jobling, 1994). Protein deposition, at 59.9 kJ/g, is energetically more expensive than lipid deposition at 43.5 and 55.3 kJ/g from lipid and non-lipid origins (Emmans, 1994), but this does not overrule the gross efficiency of protein deposition because the higher energy cost is small compared to the four- to fivefold increase in wet weight gain.

It follows that growth of protein, lipid, and structural tissues demands different amounts of energy and therefore feed, so the protein-to-lipid ratio (PLR; or derived from that, in anatomical terms: the lean-to-fat ratio) of body weight gain must also correlate to FI; see **Figure 1** where backfat depth in growing pigs represents PLR, and has genetic correlations with FI ranging from +0.38 to +0.67.

Variation in PLR is due to variation in protein content and/or in lipid content, and there is a difference between fish and the terrestrial species here. Body lipid content is highly variable in both groups, and body protein and body lean content show moderate to large genetic variation in growing pigs and cattle (e.g., Ciobanu et al., 2011; Thonney, 2015). But the protein content of wet body weight in fish shows zero or very low genetic variation; this protein homeostasis is partly controlled by FI (Shearer, 1994; Simpson and Raubenheimer, 2005; Kause et al., 2009). This lack of variation has led to a lack of interest in protein content and PLR in fish, and FE studies have mainly focused on the effect of body lipid content as such. Of course, variation in lipid content leads to variation in PLR whether protein content varies or not. In the following text we focus on PLR as the preferred high-level breeding goal trait, realizing that the actual selection trait in fish will likely be body lipid deposition – just like the common proxy trait in pigs is backfat depth as in **Figure 1**.

Third: the other obvious element is body size: larger animals need more nutrients for body maintenance. The relevant trait here is metabolic body weight (MBW), commonly calculated as body weight raised to some power that depends on animal species and stage of development. The exponent is either estimated from the data, or fixed at a default value, typically ⅔ or ¾ in endotherms (see Heussner, 1982 for a critical review). In fish, exponents of 0.79–0.82 have been used for rainbow trout, gilthead seabream (*Sparus aurata*), European sea bass (*Dicentrarchus labrax*), and white grouper (*Epinephelus aeneus*) (Lupatsch et al., 2003b; Grima et al., 2010; Kause et al., 2016).

FI in growing animals can then be estimated from the abovementioned underlying traits. A simple way would be by multiple linear regression as:

(1) $$\text{FI}={\text{b}}_{\text{GR}}\times \text{GR}+{\text{b}}_{\text{MBW}}\times \text{MBW}+{\text{b}}_{\text{PLR}}\times \text{PLR}+\text{residual}$$

The partial regression coefficients *b* represents conversion factors, quantifying how the underlying traits relate to FI. In the literatureted in the attributes of the regression equation as such. But **Figure 3** summarizes the published values of (1 -*R*^2^) for analyses like this, see section “Focal Trait 2: Residual Feed Intake.”

Such a regression carries a residual term: the proportion of FI that is not explained by the traits in the regression model. This will be due to measurement error (of any of the traits involved here), to model errors (e.g., the above linear additive model if the true state of nature is curvilinear or involves interactions), and/or to individual animal-intrinsic deviations from the population-wide conversion factors *b*. The imperfect univariate correlations of FI with GR and backfat depth in **Figure 1** illustrate the usefulness of a multivariate approach such as Model 1.

Following Model 1, the FI predictor for an individual growing animal becomes:

(2) $$\text{FI}={\hat{\text{b}}}_{\text{GR}}\times \text{GR}+{\hat{\text{b}}}_{\text{MBW}}\times \text{MBW}+{\hat{\text{b}}}_{\text{PLR}}\times \text{PLR}+\hat{\text{RFI}}$$

where RFI is residual FI: the part of observed FI that is not explained by the observed GR, MBW, and PLR of the individual, combined with the population-mean estimates $\hat{\text{b}}$ of the conversion factors. RFI was introduced by Koch et al. (1963); a positive residual implies that an individual is consuming more than predicted from its body size and its GR and growth composition (inefficient), and a negative value implies that an individual is consuming less than that (efficient).

Residual FI according to Model 1 is then:

(3) $$\hat{\text{RFI}}=\text{FI}-({\hat{\text{b}}}_{\text{GR}}\times \text{GR}+{\hat{\text{b}}}_{\text{MBW}}\times \text{MBW}+{\hat{\text{b}}}_{\text{PLR}}\times \text{PLR)}$$

As per above, RFI comprises measurement error, model error, and animal-intrinsic deviations from the population means of the *b* values. Note that Model 3 is a very simple form for RFI calculation; more elaborate forms may include elements such as the nutritional composition of the feed or health-related traits.

**FIGURE 2:**
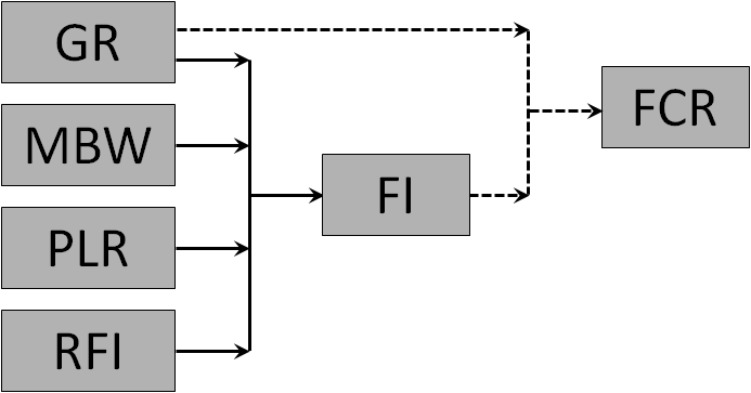
Interrelationships among feed conversion ratio (FCR; gross feed efficiency), growth rate (GR), feed intake (FI), metabolic body weight (MBW), body protein-to-lipid ratio (PLR), and residual feed intake (RFI; net feed efficiency) in growing animals. Solid arrows represent functional biological relationships; dotted arrows represent the simple mathematical equation FCR = FI/GR.

The interrelationships among the component traits of FCR are summarized in **Figure 2**. FCR is a simple mathematical function of GR and FI, and the traits underlying FI are GR, MBW, PLR, and RFI.

## Focal Traits for FCR Improvement

The aim is to improve FCR by finding alternative traits for the numerator in the FCR equation, FI. How can FI then be reduced via its underlying traits? Given Model 2, the answer is: by reducing *b*_GR_ × GR, by reducing *b*_MBW_ × MBW, by reducing *b*_PLR_ × PLR, and/or by reducing RFI. However, not all these options are sensible in practice.

A reduction of GR is not appropriate, given the fact that GR should be *increased* in order to improve (i.e., reduce) FCR: **Figure 1** shows 56 genetic correlation estimates between FCR and GR in growing pigs that range from -0.05 to -0.91 (-0.51 on average), accompanied by three suspicious positive outliers.

Metabolic body weight is largely proportional to slaughter weight. In weight-based delivery systems this is more or less fixed, and in time-based delivery systems it is strongly confounded with GR. Both these systems are used in fish farming.

This leaves PLR (or lipid or protein content separately) and RFI as the focal traits for FI reduction. They are dealt with in sections “Focal Trait 1: Body Protein-to-Lipid Ratio” and “Focal Trait 2: Residual Feed Intake,” respectively.

### Focal Trait 1: Body Protein-to-Lipid Ratio

#### Recording Protein and Lipid

The PLR of the animal body can be measured with varying levels of effort and precision. In the western world, individual slaughter pigs are commonly paid for by the abattoir based on their carcass weight and (more relevant here) on their estimated carcass lean content. Note that this is an anatomical trait, not a chemical one. Mohrmann et al. (2006) report correlations of +0.98 for protein mass versus lean tissue mass, and likewise for lipid mass versus fatty tissue mass. Lean content is commonly estimated from actual in-slaughter-line (i.e., *postmortem*) measurements of the depth or cross-section area of the loin muscle (*m. longissimus dorsi*), and of the depth of its subcutaneous fat cover. Currently, the most sophisticated high-throughput equipment is AutoFOM (Brøndum et al., 1998) which estimates carcass lean content in a few seconds with an *R*^2^ value of 70–80% (e.g., Branscheid et al., 2011).

For *in vivo* recording in performance testing of nucleus selection candidates, several ultrasound-based methods have been widely applied since the 1970s to predict body lean content in pigs, typically with *R*^2^ values around 70% (e.g., Krieter et al., 1990; Busemann et al., 1991; Johnson et al., 2004). Breeding value estimation is commonly based on such *in vivo* data combined with *postmortem* abattoir carcass measurements (as above) on relatives. Knap and Wang (2012) show that selection on this type of information (with its inherently far-from-perfect correlation to PLR) has led to considerable improvement of industry-wide FCR, as a correlated selection response.

It helps that more than half of the body lipid content of a slaughter pig is typically located in its subcutaneous fatty tissue (e.g., Knap and Jørgensen, 2000), and that the genetic correlation of subcutaneous fat measures with the second largest depot (intermuscular fat, holding about a quarter of body lipid content) is rather high at +0.4 to +0.6 (e.g., Suzuki et al., 2014); as a consequence, subcutaneous fat measures (such as backfat depth) form a good approximation of total body fat content.

Protein content recording requires laborious and expensive chemical analysis. In fish, fillet percentage can be recorded on slaughtered sibs of selection candidates, and can additionally be predicted *in vivo* from morphological measurements on the candidates, yet its prediction is challenging as reflected by modest *R*^2^ values (*R*^2^ = 0.02–0.18 for European sea bass). Compared to these phenotypic relationships, the genetic correlations between the fillet% predictors and the true fillet% are much stronger, up to 0.67 (Vandeputte et al., 2017).

In many fish species, most of the body lipid is contained in the muscles and in the viscera (e.g., Regost et al., 2001; Boujard et al., 2004), or subcutaneously in flatfish species. Muscle lipid content can be predicted both *postmortem* and *in vivo* using spectroscopy or microwave technology with reasonably high throughput and with *R*^2^ values of 0.49–0.88 (Solberg et al., 2003; Kolstad et al., 2004; Toussaint et al., 2005; Folkestad et al., 2008; Collewet et al., 2013; Brown et al., 2014; Wu et al., 2015; Janhunen et al., 2017). Lipid deposition at different parts of the body forms genetically different traits, with even negative correlations observed (Tobin et al., 2006; Kause et al., 2007), so it must be recorded at multiple body locations. In salmonids, visceral lipid content can be approximated by total viscera mass (a *postmortem* trait equivalent to abattoir lean content data on sibs in pig breeding) after a few days of fasting to release the gut content: visceral lipid is the most variable part of total viscera mass so that the proteinaceous part of intestines and liver cancel out as more or less constant (Kause et al., 2007).

#### Genetic Correlations of Protein and Lipid Content With FCR

**Figure 1** shows estimates of the genetic correlation of FCR with backfat depth in growing pigs as updated from Knap and Wang (2012, Figure 1b). These correlation estimates range from -0.15 to +0.71 (+0.27 on average). By comparison, Kause et al. (2016) report genetic correlations between body lipid content and FCR at +0.58 (total body lipid) and +0.68 (muscle lipid), and –0.39 (visceral lipid) in rainbow trout, in line with the weakly positive or even negative correlations among these lipid traits (Tobin et al., 2006). Consistent with these correlations, a trout line with low muscle lipid content showed better FE and protein efficiency than a high muscle lipid content line (Quillet et al., 2007; Kamalam et al., 2012). Quinton et al. (2007b) calculated that simultaneous selection for low lipid content and high GR in European whitefish is expected to increase the correlated genetic response in FCR compared to selection for GR only. Of course, such relationships may depend on species and age, and on nutritional and experimental settings, and hence the fattest fish may sometimes be the most efficient: Grima et al. (2010) subjected sea bass to a fasting-and-refeeding regime to quantify energetic efficiency (see the end of section “Focal Trait 2: Residual Feed Intake”) and found (non-significant) negative correlations between RFI and muscle fat content; to what extent such results can be generalized to real-life conditions is unclear.

#### Body Protein-to-Lipid Ratio: Conclusion

Body lipid mass can probably be approximated with higher precision in fish than in pigs, certainly *in vivo*; and the genetic correlation between fatness and FCR may well be stronger in fish than in pigs. Still, selection for such imperfect proxy traits in pigs has led to a very considerable industry-wide improvement of FCR, so the scope for improvement in fish looks promising.

An important side issue is to what extent a reduction of muscle lipid content would be acceptable in terms of final product quality, especially in salmonids and scombrids. Muscle lipid content typically has an intermediate optimum, but for visceral lipid a reduction is preferable (Kankainen et al., 2016). As long as good data is available, proper selection indexing is the method of choice to keep any breeding system with antagonistic elements under control. This has worked very well for decades in cattle, pig and poultry breeding; intramuscular fat content is a commercial breeding goal trait in pigs and beef cattle, to neutralize negative correlated responses of selection for reduced subcutaneous fat depth (e.g., MacNeil et al., 2010; Maignel et al., 2011). Quinton et al. (2007b) and Kause et al. (2016) provide such examples to simultaneously control growth, lipid deposition, and FCR in fish.

### Focal Trait 2: Residual Feed Intake

It follows from formula (3) that individual variation in RFI is due to deviations from the population average, caused by (i) systematic deviations from the true state of nature by the statistical model used, (ii) individual measurement errors in GR, MBW, PLR, and/or FI, and (iii) individual deviations from the population means of the conversion coefficients *b*. If RFI is to be used as a selection trait, it is essential to understand which of these factors dominate the system.

Deviations from the mean ${\hat{\text{b}}}_{\text{GR}}$ coefficient from Model 2 will mostly be absorbed by the PLR element of the model: the *composition* of body weight gain, with its metabolic consequences.

One possible source of deviations from the mean ${\hat{\text{b}}}_{\text{PLR}}$ coefficient from Model 2 would be variation in the amount of water deposited with each unit of protein. But Emmans and Kyriazakis (1995, Figure 1), Coudenys (1998, Figure 30), and Weis (2001, Figure A9) show data on physical water versus protein mass with correlations of +0.98 in growing pigs; Mohrmann et al. (2006, Figure 7) show the same as measured by D_2_O dilution, with a correlation of +0.91. Similarly, in rainbow trout, water mass and protein mass have a correlation of +0.99 (data from Kause et al., 2016). Another possible source is variation in the gross efficiencies of protein and lipid deposition, for example, because of variable protein turnover rates; but Knap (2008) shows that such deviations likely explain less than 5% of the total variance of the maintenance requirements of growing pigs.

It follows that, in pigs, the dominating animal-intrinsic element in the residual term should be due to individual deviations from the mean ${\hat{\text{b}}}_{\text{MBW}}$ coefficient from Model 2. The conventional way of quantifying maintenance requirements is by, say, 600 × BW^0.75^ kJ/day in growing pigs or 50 × BW^0.80^ kJ/day in subadult salmonids – and as with every other parameter in biology we must expect the *b*_MBW_ values of 600 or 50 (and the 0.75 or 0.80 power values: estimates of the two are usually strongly confounded) to vary among individuals. Such variation is due to differences between individuals in basal metabolic rate, thermoregulatory functions, immune response functions, physical activity, and responses to social factors (reviewed by Knap, 2008; Knap and Wang, 2012; Gilbert et al., 2017).

**FIGURE 3:**
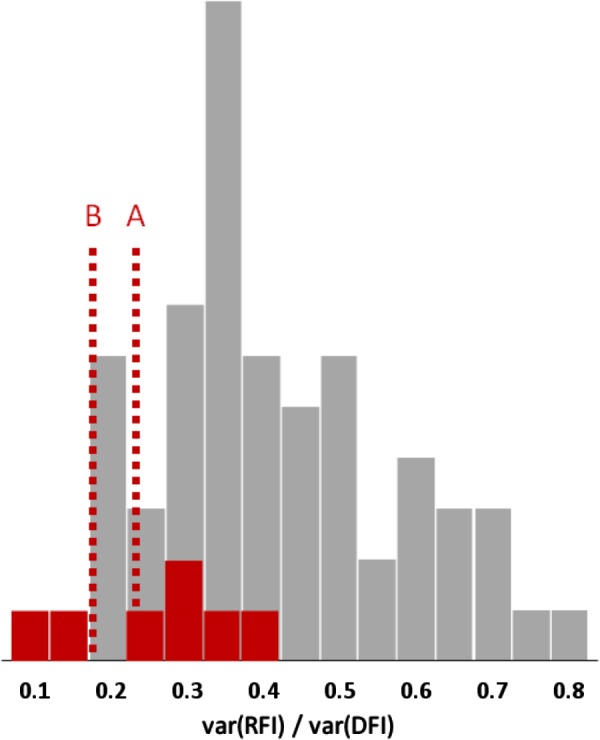
Frequency distributions of the proportion of feed intake (FI) variance explained by variation in residual feed intake (RFI) in mammals or birds (gray bars) and in fish (red bars); 61 estimates from 39 literature sources. The red reference lines indicate the possible mean values for fish, after adjustment for the absence of body fatness traits in the RFI model (A) and for possibly stronger correlations between body fat content and feed efficiency in fish than in mammals and birds (B).

So, when body composition is included into the model, RFI covers an aggregate of metabolic traits that explains 18–80% of the phenotypic variance of FI in mammals and birds; **Figure 3** shows 61 estimates of this variance component, with an average value of 40%. By contrast, studies in gilthead seabream, African catfish (*Clarias gariepinus*), rainbow trout, sole (*Solea solea*), and Nile tilapia (*Oreochromis niloticus*) (Doupé and Lymbery, 2004; De Matos Martins, 2005; Mas-Muñoz et al., 2011; Kause et al., 2016; De Verdal et al., 2017b) have produced seven estimates where RFI explains 13–40% (25% on average) of the phenotypic variance of FI.

Note that imperfect measurement of chemical PLR via its anatomical proxy lean-to-fat ratio (which is itself imperfectly measured by the various ultrasound and other methods) leads to measurement error variation that will be absorbed by the residual term of models such as Model 1. In other words, in pigs RFI usually includes an PLR-related component. When PLR is not included in the model at all, its entire variation will largely end up in the RFI variance. This is particularly relevant because PLR was not included in any of the abovementioned fish studies, so those 13–40% RFI variance components are systematically overestimated. The results of Labroue et al. (1999, **Table 1**) can quantify this impact; they evaluated alternative RFI models in growing pigs, with and without subcutaneous backfat depth added to the regression simultaneously with GR and/or MBW. The addition of fat depth to these models reduced the RFI variance components by proportionally 0.13–0.21. Applying that same reduction to the above-mentioned fish results leaves RFI variance components of 10–35% of the variance of FI; it would therefore be useful to perform similar analyses on farmed fish data.

The genetic correlation between fat content and FCR seems to be higher in fish (+0.58 to +0.68 in rainbow trout as from Kause et al., 2016) than in growing pigs (around +0.27 as from **Figure 1**) – judging from these values possibly more than twice as high. Therefore, the above-mentioned reduction of the RFI variance component in pigs by proportionally 0.13–0.21 may well be at least twice as strong in fish – and that would leave RFI variance components of, say, 7–30% of the variance of FI: less than 20% on average (see **Figure 3**).

Recall that this variation would largely be due to individual deviations from the above mean population ${\hat{\text{b}}}_{\text{MBW}}$ coefficient of 50 kJ/day per BW^0.80^ from Model 2. Measuring such deviations explicitly, rather than inferring them from models such as Model 3, might offer a more effective (but also more expensive) way to improve efficiency; Knap (2000) studied such deviations as caused by variation in body protein turnover rates and in thermoregulatory processes in growing pigs.

**FIGURE 4:**
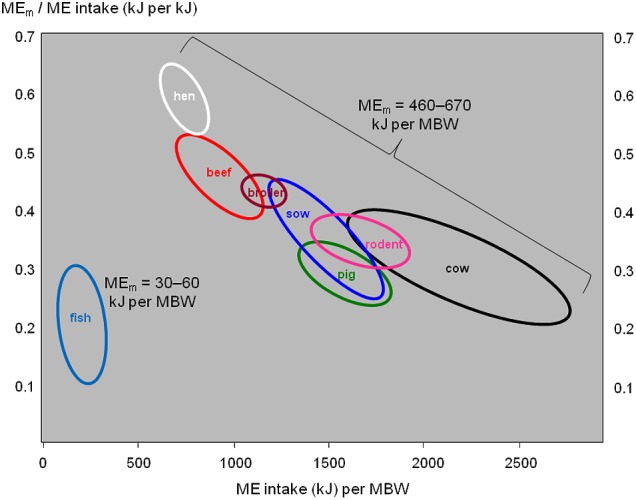
Daily metabolizable energy (ME) requirements for body maintenance (ME_m_) in relation to daily ME intake in various fish, mammal, and bird species. MBW, metabolic body weight in kg^β^ (typically, 0.6 ≤ β ≤ 1). Data for fish from Gatlin et al. (1986), Rodehutscord and Pfeffer (1999), Lupatsch et al. (2003a,c), Azevedo et al. (2005), Lupatsch and Kissil (2005), Lopez and Leeson (2008), and NRC (2011). Data for endotherms from NRC (1995, 2000, 2001, 2012), Reyes et al. (2011), Sakomura (2004), and Thiele and Pottgüter (2008).

The maintenance requirements of fish (as cold-blooded poikilothermic animals) are much lower than in mammals or birds of the same body weight (Brett, 1972). **Figure 4** illustrates this: the endotherm species show a wide range of energy intake per unit of MBW (horizontal axis), and an equally wide range of the proportion of that energy intake that is devoted to body maintenance (vertical axis), but their maintenance requirements are relatively similar around 550 kJ per unit of MBW. By contrast, fish consume much less energy than the endotherm species do (horizontal axis), and the proportion of it devoted to body maintenance (vertical axis) is around the lower limit of the endotherm range; maintenance requirements range around 45 kJ per unit of MBW.

Hence RFI, as an indicator of metabolic costs, explains a lower amount of the variation in FI in fish than in endotherms.

The relationship between metabolic maintenance costs and FCR has been studied in fish using weight loss during fasting as a proxy of the costs. During fasting, fish use their internal energy sources to cover maintenance costs, so the weight loss may serve as an indicator. Grima et al. (2008, 2010) showed that weight loss during fasting and weight gain during re-feeding are correlated with FE in rainbow trout clones and in sea bass. However, the results from other studies (Daulé et al., 2014 on sea bass; De Verdal et al., 2017a on Nile tilapia) are inconclusive, showing no relation between weight loss and FCR.

There would be a need to quantify the variation in FI and FCR explained by each of the component traits of **Figure 2**, to obtain solid values for the contribution of each component trait. Such trait contributions will likely differ between fish versus endotherms, and between growing meat-type animals versus mature dairy cattle, due to increasing maintenance costs when moving from fish via growing meat animals to dairy cattle (**Figure 4**).

#### Residual Feed Intake: Conclusion

It follows that, on average, the proportion of the variance of FI that is covered by variation in maintenance requirements (as approximated by RFI) is likely only half as large in fish as in mammals or birds. Improvement of RFI (net FE) is then a much less effective and less efficient way to improve FCR (gross FE) in fish than it is in mammals or birds. This is particularly relevant because estimation of RFI (e.g., with Model 3) requires FI recording.

## Discussion

Improvement of gross FE (FCR) in growing fish can be (and has been) accomplished by selection for increased GR. But the correlation of growth with FE is typically only modest (see **Figure 1** for published values in growing pigs), and hence there is room for further improvement of FE through methods other than growth selection. We propose that the most effective additional methods are selection for reduced body lipid content and for reduced RFI. Both methods require more or less sophisticated recording equipment, and in particular the estimation of RFI requires recording of FI which remains to be challenging.

In breeding programs for mammals and birds, both these approaches can be very effective. Despite the high costs of FI recording, the RFI approach can be cost-efficient because maintenance requirements are high and therefore RFI variation covers a large part of the FI variance. Maintenance requirements of fish are lower and therefore RFI variation covers a much smaller part of FI variance; and accurate and high-volume routine individual FI recording is much more challenging in fish than in mammals or birds. In terms of FE improvement, fish breeding can then benefit from the pig breeding experience in two ways: first, by adopting the notion of routinely recording the body PLR and implementing it as a serious breeding goal trait; and second, by evaluating the various options to quantify net FE in terms of RFI, and the relevance of including this trait in the breeding goal.

We propose that selection for reduced body fat content is likely a more effective way to improve FCR in fish than selection for reduced RFI, and certainly a more cost-efficient way to do so; cost-efficiency is usually the main restriction to genetic improvement in any commercial breeding program. As long as body fat content is dealt with as an explicit selection criterion, the only valid reason for FI recording would be the requirement for RFI reduction. So, if RFI reduction is not required (because it is not effective enough), then there would be no need for the expense and effort of individual FI recording – and in fish breeding this would be a very desirable situation.

## Recommendations

Clearly, much of the above is a generalization of the true state of nature, with a need for clarification which requires more data on the relevant issues. This leads to focus for future R&D needs:

(1) Quantification of the proportion of FI and FCR variation explained by the component traits growth, body composition, and body maintenance. The amount of variation explained by the different component traits will likely differ between leaner (younger) and fatter (older) fish, between low and high-fat fish species, and between different diets and environments. For the results to reflect growth under commercial conditions, such exercises need to be done in group housing as individual housing does not properly reflect commercial conditions (in fish: Øverli et al., 1998; in pigs: Knap, 2009). Fish density and separate versus mixed-family rearing are known to induce genotype-by-environment interactions in growth (Vandeputte et al., 2009; Sae-Lim et al., 2016). Yet, feed utilization recorded on individually kept fish is independent of potential social interactions that may reduce performance of socially sub-ordinate fish, or conversely, high density may elevate appetite induced by group feeding (Øverli et al., 1998).
(2) Rapid, cheap, and accurate non-destructive recording methods for body lipid content need to be validated for the main farmed fish species, and proven to be functional in routine breeding under commercial conditions. An example of such a validation approach was provided by Janhunen et al. (2017).
(3) More estimates for the correlations of body lipid content with gross and net FE are needed. Naturally, this can only be achieved with FI recording, but this recording does not need to become perpetual routine as it will have to be for body fat content. What is needed is datasets of sufficient info-quality to allow for accurate estimation of the correlation coefficients.
(4) From Model 3 it follows that a proper FI recording protocol is needed. Given the current laborious methods, the development of (semi)automated methods is appealing and may allow recording of a long-term FI.
(5) Test the use of family tanks to record FI of sib groups. Some breeding programs have hundreds of small or moderate-sized family tanks that could be used for recording FI at the group level. Such tank-based FI records can pragmatically be combined with individual-level weight gain and lipid deposition recorded *in vivo*. This could provide a practical way for genetic improvement of FE. Each family can be held in a tank, or replicated tanks, but statistical methods exist to estimate breeding values under tank-based trait recording when the families are mixed within a tank (Peeters et al., 2013).
(6) Development of genomic selection methods when the reference population has phenotypes on growth, lipid deposition, and FI. FI could potentially be measured at the family mean-level, but then there is a need to develop methods on how to apply genomic selection based on family means, or repeated family-mean records. Genomic selection could also be developed based on individual recording of FI if a suitable phenotyping method is available, as suggested in point (3).
(7) A detailed focus on the components of growth leads to “precision breeding.” Protein retention efficiency is the ultimate efficiency trait to be improved. A fish can assemble its body protein only from amino acids in the feed, and high-quality proteins are among the most expensive ingredients of aquafeed, often of limited supply. The current evidence on rainbow trout in fact indicates that restricting lipid deposition to improve FCR also improves protein retention (Kause et al., 2016). More work is needed on the genetics and genomics of lipid and protein growth and retention, to understand the potential for their genetic improvement. One line of thinking is that selection for FCR may generate a re-allocation of resources from, e.g., the immune system to body growth. So, selection for better digestibility of nutrients would be an alternative option as it would improve the absorption of nutrients without changing their partitioning.
(8) Fish form an important source of healthy polyunsaturated fatty acids (PUFA), including n-3 fatty acids like omega-3. There is evidence that lipid content in fish muscle is negatively associated with the PUFA content in muscle. Moreover, in farmed fish the important n-3/n-6 fatty acid ratio is often suboptimal due to high amount of total lipid, and the increased use of vegetable oils in feeds of carnivorous fish (Cronin et al., 1991; Strobel et al., 2012). Fatty acid composition of farmed fish in general may hence develop unfavorably with strong selection for improved growth when muscle lipid content is not under control. There is a need to understand the ways to improve and control fatty acid retention and content to maintain nutritional quality, when simultaneously modifying body lipid content.

## Author Contributions

PWK wrote the first draft, including the information on pig breeding and genetics, the information on energy metabolism, some of the information on fish genetics, and the discussion and conclusion. AK provided most of the information on fish breeding and genetics, and completed the discussion and conclusion.

## Conflict of Interest Statement

PWK is employed by company Genus-PIC. The other author declares that the research was conducted in the absence of any commercial or financial relationships that could be construed as a potential conflict of interest.
